# A survey of cancer patients’ interest in undertaking exercise to promote relaxation during radiotherapy for breast cancer and metastatic cancer

**DOI:** 10.1186/s13014-024-02459-w

**Published:** 2024-05-31

**Authors:** Rebecca Moser, Nina A. Mayr, Jana Nano, Sophie T. Behzadi, Sophia Kiesl, Stephanie E. Combs, Kai J. Borm

**Affiliations:** 1 Department of Radiation Oncology, TUM School of Medicine and Health, Technische Universität München (TUM), Klinikum rechts der Isar, Ismaninger Straße 22, 81675 Munich, Germany; 2 School of Human Medicine, Michigan State University, East Lansing, MI USA; 3 Institute of Innovative Radiotherapy (iRT), Department of Radiation Sciences (DRS), Helmholtz Zentrum München, Ingolstädter Landstraße 1, 85764 Oberschleißheim, Germany; 4 German Consortium for Translational Cancer Research (dktk), Partner Site Munich, Berlin, Germany

**Keywords:** Radiotherapy, Relaxation exercises, Curative and palliative treatment, Active involvement to radiotherapy

## Abstract

**Background:**

Approximately 25–50% of patients undergoing radiotherapy (RT) experience psychological distress and anxiety, which can detrimentally affect both their quality of life and treatment outcomes. While previous research has demonstrated that relaxation exercises can enhance the tolerability of RT and alleviate associated stress and anxiety, the specific needs for such therapies in radiation oncology remain under-explored. This study aims to investigate the demand for and preferences toward relaxation exercises among radiotherapy patients, addressing a critical gap in patient-centered care.

**Methods:**

A prospective pseudonymized survey study using a one-time paper-based questionnaire was conducted from 2022 to 2023 among patients undergoing curative-intent RT for breast cancer or patients undergoing palliative RT for bone metastases. Patients were asked in a 11-item questionnaire about their anxiety, pre-existing practice of relaxation exercises/interventions, their interest in relaxation exercises, and preferences on the type and format of instruction. Data were analyzed descriptively.

**Results:**

100 patients (74 female and 26 male) responded, of whom 68 received curative-intent adjuvant RT and 32 palliative RT. Median age was 62 years. 78% of patients indicated a desire to be actively involved in their radiotherapy, but only 27% had used relaxation exercises prior to RT. 44.8% of both curatively and palliatively treated patients who wanted to be actively involved in their therapy desired to learn how to best relax. 56.4% of respondents were willing to spend extra time learning offered exercises.

**Conclusion:**

The survey indicates that patients undergoing RT, both for curative or palliative intent, desire relaxation exercises to relieve stress and anxiety from RT. It is therefore important to assess the need for relaxation interventions in individual patients and to develop suitable programs or collaborate with other healthcare professionals to meet these needs.

**Supplementary Information:**

The online version contains supplementary material available at 10.1186/s13014-024-02459-w.

## Introduction

Radiotherapy (RT) is a central component of cancer treatment in the curative intent, e.g. as commonly practiced adjuvant therapy for breast cancer patients, and also in the palliative setting [1]. It is estimated that between 25% and 50% of patients undergoing RT experience significant psychosocial distress and anxiety [2–5]. Previous studies highlight that these emotional responses are often triggered by concerns of treatment procedure including loss of control, resentments of radiation exposure and uncertainties regarding the oncologic outcome and potential side effects of radiotherapy [6–13].

As RT procedures have become much more complex and increasingly require active participation by the patient, stress and anxiety become greater obstacles towards treatment compliance and effective patient cooperation in their RT.

Stress and anxiety do not only lower patients’ quality of life during and after the treatment but can also have a negative effect on the treatment success [14, 15]:

1. In the curative setting of adjuvant RT for breast cancer the patient is actively involved. An important example for the need for such active patient cooperation in RT is the deep inspiration breath hold or procedure (DIBH). During the DIBH procedure the patient is required to hold the breath daily for prolonged time periods under strict on-demand timing while the radiation beams are delivered. DIBH is frequently used in RT for breast cancer, one of the most commonly practiced RT procedures. DIBH is critically important to reduce the radiation dose to the heart in RT for breast cancer, so that the probability of well-known cardiac morbidity and mortality can be reduced [16]. There is evidence that anxiety has a direct effect on breathing patterns, which may reduce the capacity to perform deep inspiration breath hold resulting in suboptimal radiation dose reduction to the heart [17–21].
2. In the palliative setting of bone metastases the most frequent indication for RT is pain [22–26]. The perception of pain is extremely complex and not only a sensory expierence but involves a wide range of biological, psychological and social factors [27, 28]. Treatment related stress can therefore interfere with the actual treatment aim of reducing pain.

Despite ample reports of distress and anxiety in RT, the existing literature regarding prevention and treatment of anxiety in RT is sparse. The few existing studies, however, consistently show that relaxation training including yoga, listening to music and progressive muscle relaxation can have a positive effect on reducing distress and anxiety during RT [29–33]. Yet, structured programs for addressing distress and anxiety in radiotherapy are largely missing in routine clinical practice. Given the high prevalence of distress and anxiety among these patients, a substantial need for such interventions is expected. As a first step to address this dilemma, assessment of the need for interventions to prevent and/or reduce RT-associated stress and anxiety is required.

The current study aimed to systematically quantify the need for relaxation exercises among patients undergoing RT. By conducting a survey, we seek to assess not only the level of desire among these patients to learn relaxation exercises to alleviate RT-related anxiety but also their willingness to dedicate additional time to such practices. The survey will further explore patient preferences concerning the types of relaxation interventions they favor, as well as their preferred formats and settings for training. Given the heightened relevance of these considerations for patients actively involved in their treatment and those experiencing significant pain, this study specifically focuses on two distinct patient cohorts: those undergoing curative RT for breast cancer, as well as those receiving palliative RT for bone metastases. This dual focus is intended to cover a broad spectrum of patient experiences and needs associated with RT, which are crucial for developing comprehensive patient-centered care strategies.

## Methods

### Study design

This prospective, pseudonymized survey was conducted from June 2022 to March 2023 at the Department of Radiation Oncology, Technical University of Munich, Germany. The survey, comprising a one-time paper-based questionnaire, was administered to patients in the waiting room during their scheduled daily radiation therapy sessions [34].

### Eligibility and participants

Participants included adults aged 18 years or older who were undergoing either adjuvant RT for breast cancer or palliative RT for bone metastases from different solid tumors. The inclusion of these two patient groups were designed to enhance the generalizability of the findings and provide a more holistic view of the needs across different cancer care scenarios. Patients under the age of 18 are generally considered minors and require parental consent to participate in research, which can complicate the consent process and introduce additional ethical concerns. Moreover, children and adolescents may have different developmental and psychological responses to illness and treatment, which could confound the results intended for an adult population. Eligibility criteria required patients to be proficient in German and capable of physically and mentally completing a structured questionnaire. Physicians approached all eligible patients during the study period to request their participation, with each consenting patient completing the questionnaire while awaiting treatment.

The study was approved by the local institutional review board (2022-321-S-NP), and all participants provided written informed consent. This survey is part of a project investigating relaxation and breathing training in breast cancer patients funded by the German Cancer Aid (Deutsche Krebshilfe).

### Questionnaire development and content

To address the specific research objectives, we developed a questionnaire in collaboration with oncology and psycho-oncology experts, aiming to capture the unique aspects of anxiety and relaxation needs directly related to the radiotherapy experience. While validated scales like the HADS and STAI are well-suited for general clinical use, our tailored questionnaire allowed us to delve deeper into the specific concerns and therapeutic needs of our patient population.

We used a structured questionnaire consisting of 11 questions with 12 subitems, of which 9 items were scaled answers on a four-point scale and two items had multiple choice answers. The study was designed to collect data on various aspects, including anxiety of RT compared to other cancer-specific therapies and pre-existing relaxation practices. Patients were asked about their motivation to actively participate in their treatment and their willingness to spend time on training, as well as their desire for relaxation therapy. Furthermore, the preferred mode and setting as well as the type of relaxation training were queried. The questionnaire is provided in Supplementary Appendix A1.

### Statistical analysis

The collected data were pseudonymized and analyzed using SPSS version 26.0. We conducted descriptive statistical analyses to present categorical data as counts and percentages. Comparative analyses between the curatively and palliatively treated patient groups were performed separately to ascertain differences in needs and responses towards relaxation practices. Results were visually represented through various graphical formats to enhance the clarity of the findings.

## Results

A total of 100 patients (of 100 invited patients) responded to the study. Of these, 74 were female and 26 were male, with a median age of 62 years. Among the respondents, 68 females received adjuvant RT for breast cancer, and 32 patients (6 females, 26 males) were treated with RT for bone metastases. 24 patients (5 patients with breast cancer, 19 with palliative RT; 9 females/15 males) had a history of previous RT.

### Curative-intent radiotherapy for breast cancer

**Fig. 1:**
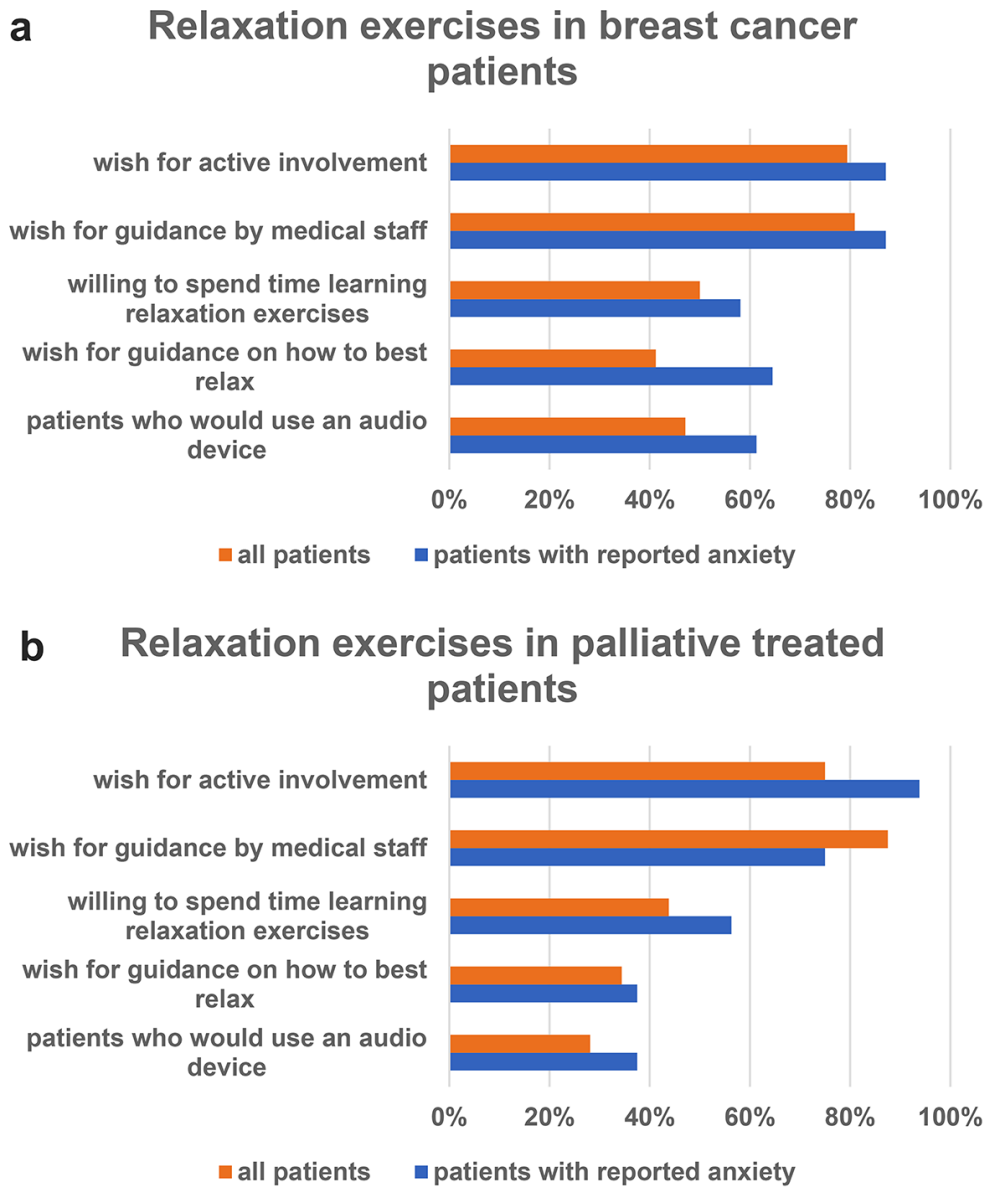
Willingness for and preferences on format and timing of relaxation exercises. (**a**) shows data for breast cancer patients receiving adjuvant radiation, and (**b**) shows data for patients treated palliatively for bone metastases. Data are presented for patients with (blue columns) and without (orange columns) pre-RT anxiety (queried by the questionnaire)

Among the 68 breast cancer patients treated with adjuvant curative-intent RT, the majority (79.4%, *n *= 54/68) desired to be actively involved in their RT in order to contribute to the best possible therapy success, but only 30.9% already practiced relaxation exercises before RT (Fig. 1a). A desire to learn relaxation exercises to relax before and during RT was expressed by 41.2%. To assess patients’ overall motivation to engage in relaxation exercises, we asked about their willingness to invest time to learn relaxation exercises and found that 50% were willing to spend time in learning relaxation exercises. Most wished to be guided by medical staff for this purpose (*n* = 55, 80.9%).

Patient preferences for specific types, formats and environments for relaxation training were examined. Relaxation training at home was preferred by 55.9%, but a substantial proportion desired exercises in the waiting room and treatment room. Among the various relaxation exercises, breathing exercises had the greatest appeal (39.7%), followed by progressive muscle relaxation (32.4%) and relaxation music (30.9%, Table 1a).

**Table 1:** Preferences regarding the location and method for relaxation exercises in radiotherapy

	a) Adjuvant breast cancer RT (*n* = 68)		b) Palliative Treatment (*n* = 32)		Total (*n* = 100)	
**Relaxation exercises preferences based on location**						
	**Yes** ***n*(**%**)**	**No** ***n*(**%**)**	**Yes** ***n*(**%**)**	**No** ***n*(**%**)**	**Yes** ***n*(**%**)**	**No** ***n*(**%**)**
In the treatment room	23 (34)	45 (66)	9 (28)	23 (72)	32 (32)	68 (68)
Before the RT in the waiting room	10 (15)	58 (85)	7 (22)	25 (78)	17 (17)	83 (83)
At home	38 (56)	30 (44)	11 (34)	21 (66)	49 (49)	51 (51)
**Preferred method of relaxation exercises**						
Autogenous training	15 (22)	53 (78)	11 (34)	21 (66)	26 (26)	74 (74)
Progressive muscle relaxation	22 (32)	46 (68)	10 (31)	22 (69)	32 (32)	68 (68)
Yoga	20 (29)	48 (71)	5 (16)	27 (84)	25 (25)	75 (75)
Breathing training	27 (40)	41 (60)	13 (41)	19 (59)	40 (40)	60 (60)
Dream journey/meditation	15 (22)	53 (78)	7 (22)	25 (78)	22 (22)	78 (78)
Relaxation music	21 (31)	47 (69)	10 (31)	22 (69)	31 (31)	69 (69)

Numbers in parenthesis represent percentages of count data. RT, radiotherapy

#### Patients desiring active involvement in their RT

Among the subgroup of breast cancer patients who desired active involvement in their RT (*n* = 54), the proportion of patients with pre-existing use of relaxation exercises was 29.6%, similar to that of the 30.9% of the overall breast cancer patient group. The proportions of patients who desired relaxation training (46.0%) and motivation to spend time on training (57.4%) were also overall similar. The proportion of patients who wanted instructions by medical staff increased to 94.4% (*n* = 51).

#### Patients with radiotherapy associated anxiety

The group of 68 breast cancer patients was further analyzed for the presence of pre-RT anxiety (patients, who reported being anxious in the questionnaire) and its potential influence on patients’ preferences regarding relaxation training. Nearly half (45.6%) reported experiencing anxiety before their first RT. Breast cancer patients with reported anxiety were more interested in relaxation exercises (*n* = 20/31, 64.5%) than patients who did not report any anxiety (*n* = 8/37, 21.6%). The proportion of preexisting experience with relaxation exercises practice trended higher among the breast cancer patients with reported anxiety (45.2% versus 18.9% in patients who did not report any anxiety); 64.5% desired guidance on how to best relax; and 61.3% found a digital audio tool (e.g. an app or MP3) with breathing and relaxation training helpful for self-exercise (Fig. 1a).

### Palliative radiotherapy for bone metastases

Among the 32 patients who were treated palliatively for one metastasis, 75% desired active involvement in their RT to contribute to the best possible therapy success, and 28 (87.5%) desired guidance by medical staff. One third of patients (*n* = 11/32, 34.4%) desired training in relaxation exercises (Fig. 1b). Only 18.8% (*n* = 6/32) of the palliatively treated patients already practiced relaxation exercises.

Patient preferences for formats and settings of relaxation training were overall evenly distributed among the groups of palliatively treated patients. A digital audio device (app or MP3) for a self-exercise program was preferred by 28.1%, and small group training for relaxation exercises by 25%. One third (34.4%) preferred relaxation training at home, 21.9% pre-RT in the waiting room and 28.1% in the treatment room. Among the types of relaxation practice, 13 (40.6%) were interested in breathing training, 11 (34.4%) in autogenous training, 10 (31.3%) in progressive muscle relaxation and 10 (31.3%) in relaxing music (see Table 1b).

#### Patients desiring active involvement in their RT

In 10 patients (41.7%) of the 24 palliatively treated patients with a desire for active participation in their RT, a wish for learning relaxation exercises was identified (half of them with prior RT), and 54.2% (*n* = 13/24) were willing to spend time on relaxation training (patients with prior RT: *n* = 8). Only 25% (*n* = 6) already used relaxation exercises, two of them with prior RT in their lives. All patients with a desire for active participation were interested in instructions from medical staff (*n* = 24, 100%).

#### Patients with radiotherapy associated anxiety

The wish to be actively involved was higher in patients with reported anxiety (in the questionnaire) than in patients who did not report any anxiety (93.8% vs. 75.0%). The percentage of patients who would like to be offered relaxation exercises was similar between patients with reported anxiety (*n* = 6 of 16, 37.5%) and not reported anxiety (*n* = 5/16, 31.3%). More than 40% of patients with reported anxiety would be willing to spend additional time learning relaxation exercises (Fig. 1b), compared to 31.3% of patients with not reported anxiety.

## Discussion

This study sought to establish the level of need for relaxation interventions directly from RT patients’ perspective through a survey. Our survey study addresses the current knowledge gap of patient-perceived need for relaxation exercises in more than one third of patients. Almost 80% of patients expressed a desire to actively participate in their radiotherapy, but only 27% had practiced relaxation exercises before RT. The availability of such information, which is currently lacking, can serve as a first step towards developing RT patient-specific relaxation interventions and programs in radiation oncology.

### Overall desire for relaxation techniques

Our results show a high demand for relaxation exercises among RT patients. More than one third (39%) of patients showed interest in relaxation exercises prior to and/or during RT to counteract the stress and anxiety. Almost half (48%) were willing to spend additional time learning relaxation exercises. This willingness by patients to invest additional time – in addition to the other demands and time commitments from their cancer treatment – attests to their high motivation for learning and practicing relaxation interventions. The high demand for relaxation exercises in radiation therapy can be explained by the mostly passive role of the patient, who undergoes stressful treatment in often confining immobilization and with high demands on patient cooperation, while being fully conscious (unlike in other therapies such as surgery). Our findings therefore suggest that the current lack of defined relaxation intervention programs constitutes an unmet need for RT patients.

### Specific groups: active involvement

We explored which specific patient characteristics were associated with a desire for relaxation exercises because this understanding may serve to focus efforts on specific patient groups. We found that demand was particularly high in patients with reported anxiety and those who sought active involvement in their oncologic care to contribute to the best possible success of their therapy.

Over 40% of patients who wished for active involvement in their therapy (actively care-involved patients) desired relaxation exercises. This is a substantial proportion considering that over three fourth of all patients desired to be actively involved. Our survey also revealed that only one third of actively care-involved patients already used relaxation exercises. This high interest among RT patients and the low proportion of pre-existing relaxation exercise practice underscores the current lack of availability and the need for developing relaxation interventions tailored to RT patients.

### Specific groups: anxiety

The presence of RT-associated reported anxiety had a strong impact on patients’ need for relaxation exercises. Specifically, our breast cancer patients with reported anxiety before RT frequently reported interest in relaxation exercises (65%, Fig. 1a). This finding is not unexpected, considering the known efficacy of relaxation exercises in alleviating anxiety [35–37]. This finding also emphasizes that assessment of baseline distress level may be an important criterion for identifying patients with the greatest need for training in relaxation techniques [2, 3, 7]. Overall baseline anxiety was present in nearly half (45.5%) of the breast cancer group, further underscoring the overall need for relaxation training.

Conversely, we could not identify a clear correlation between anxiety and the demand for relaxation techniques in palliatively treated patients. This may be explained by a longer experience with cancer or pain preventing motivation for relaxation exercises but can also be related to the smaller size of our palliative group (*n* = 32) and resulting inability to discern a difference.

Ample evidence shows that relaxation therapy interventions are effective in reducing stress and anxiety that are frequently associated with RT. Nunes et al. randomized 34 breast cancer patients to a group with relaxation and visualization therapy simultaneously to their RT versus a control group without any intervention. They found that relaxation and visualization therapy reduced stress, anxiety, and depression scores [38].

In a larger randomized trial by Krischer et al., 310 US patients received self-administered stress management training while undergoing RT versus standard care. In the interventional arm, patients received instructions on a videotape, an audiotape and a booklet, by which they were encouraged to exercise by themselves with instructions on breathing, active relaxation and positive thinking. While the *overall* groups showed no difference, patients with initially higher psychological distress showed reduced anxiety and depression 3 weeks after RT start compared to the pre-RT baseline with self-administered stress management training compared to standard care [39]. Krischer et al.’s findings are important in the context of our observation that patients with higher anxiety had higher demands for relaxation training (Fig. 1a), by showing that these patients also derive a higher benefit from relaxation practice [39].

### Types of relaxation exercises

We further sought to determine which specific relaxation exercises were desired by patients. The term “relaxation exercises” comprises a large variety of techniques, which vary considerably in clinical application and outcomes. A study conducted in the UK showed that music therapy positively influenced anxiety levels of patients in a waiting room for RT [40]. In a study by Siqueira et al. use of an audio sequence with a 15-minute relaxation session (including respiratory movements and nature sounds 3x/week during RT) improved quality of life evaluated by the QLQ-C30 in breast cancer patients undergoing RT [41].

A large proportion of patients in our study (> 40%) expressed interest in breathing exercises. This finding may be, in part related to the common practice of breathing maneuvers. However, preferences for other techniques, including progressive muscle relaxation, music and autogenous training (each > 30%, Table 1), were also seen, indicating that patients’ needs for specific relaxation interventions may be far more complex and require further study with respect to the specific disease status and RT delivery techniques.

### Current lack of relaxation exercises, causes, needs, possible solutions

Despite the evidence suggesting that patients benefit from relaxation exercises during RT [38, 42], there is a lack of structured offerings and implementation in clinical RT practice.

An online survey on the provision of overall complementary and alternative medicine (CAM) by Kessel et al. showed that only 32.2% of the radio oncologists and gynecologists surveyed offered overall CAM in their daily clinical practice. Relaxation interventions, however, played a minor role in the CAM interventions, which were mostly restricted to sports activities, dietary supplements and nutritional counselling [43], with much less emphasis on relaxation exercises. This data suggests a significant undersupply of these readily available interventions in clinical practice. Lack of awareness about patients’ needs or insufficient knowledge about relaxation techniques and their implementation may be reasons for the lack of offering RT-specific relaxation interventions.

Despite the availability of many free relaxation resources, our findings show that patients don’t often practice relaxation, even though they want to be actively involved in their care. This highlights a strong need for structured relaxation training designed specifically for radiation therapy as well as better implementation into daily RT practice. Currently, concepts that integrate relaxation therapy and RT are largely lacking, and this hampers patient education and patient access to RT-specific relaxation training.

Development of specific programs for patients undergoing RT, offered by radiation oncologists or through close interdisciplinary collaboration with professionals such as psychosomatic therapists, can address this need. Such programs require close tailoring to the specific disease situation and type of RT.

Our findings identified breast cancer patients undergoing adjuvant RT as a major target group for relaxation interventions. The estimated 80% need for active cooperation during RT, the interest in learning relaxation exercises by nearly half of our breast cancer patients, and the high incidence of breast cancer (as the most prevalent malignant tumor in women [44]), all suggest that breast cancer patients constitute a significant patient population to benefit from relaxation therapy alongside their RT.

We investigated two common clinical situations: curative-intent adjuvant therapy for breast cancer and palliative therapy for bone metastases.

While their specific needs and preferences varied (Fig. 1; Table 1), overall, *both*, curatively treated breast cancer patients and palliatively treated patients with bone metastases, were interested in active participation and desired guidance on how to best relax (Fig. 1). This again underscores the need to develop targeted relaxation training approaches that are tailored to specific individual patient populations and specific RT regimens, to alleviate patients’ stress and anxiety while improving the delivery of RT.

It should be noted that most patients would prefer to perform relaxation exercises at home instead of in the waiting room or during the treatment.

Our study has the general limitations of survey research, including respondent rate, nonresponse, and conformity bias. The survey was pseudonymous to mitigate nonresponse and conformity bias and was kept short to enhance the response rate. In this initial study we only addressed two clinical disease and treatment scenarios: adjuvant therapy for breast cancer (curative intent) and RT for bone metastasis (palliative intent), which are common in radiation oncology. Patients’ preferences and requirements may differ for other diseases and clinical situations, and this requires further study. Furthermore, the palliative radiation group (*n* = 32) was smaller than our curative-intent treated group, and this may reduce validity of our results. However, the results of our study can be considered hypothesis-generating for further larger studies. An important limitation to our study is the use of a short and un-validated questionnaire. Our aim was to integrate questions that directly relate to the radiotherapy experience, including specific fears, concerns, and the desire for relaxation exercises that are pertinent to this treatment modality. We designed the questionnaire in collaboration with clinical experts in oncology to ensure that the items were relevant and appropriate for the radiotherapy context. Further studies on the topic should also include standardized and validated questionnaires for measuring anxiety and depression.

## Conclusion

More than one third of patients undergoing radiotherapy wish to receive guidance on how to effectively use relaxation exercises during their treatment. For patients with a wish for active involvement the percentage increases to over 40%. A significant number of these patients desire to utilize relaxation exercises not only at home, but also in the treatment and the waiting room. These findings are largely relevant as they are widely unknown to radiation oncologists and are not taken into consideration during treatment. Our results emphasize that the entire oncology care team—comprising radiation oncologists, nurses, psycho-oncologists, and support staff—plays a vital role in assessing patient needs, implementing interventions, and monitoring their effectiveness, underscoring a collaborative approach to patient care.

### Electronic supplementary material

Below is the link to the electronic supplementary material.

Supplementary Material 1

## Data Availability

The datasets analyzed during the current study are available from the corresponding author upon reasonable request.

## Abbreviations

RT: Radiotherapy
CAM: Complementary and alternative medicine
DIBH: Deep inspiration breath hold
