# Supplementary material for: A survey of cancer patients’ interest in undertaking exercise to promote relaxation during radiotherapy for breast cancer and metastatic cancer
Source: Radiat Oncol. 2024 May 31;19:68. doi: 10.1186/s13014-024-02459-w (PMC11143565; doi:10.1186/s13014-024-02459-w)
Supplement: Supplementary file 1 — Supplementary Material 1 [file 13014_2024_2459_MOESM1_ESM.docx]

**Questionnaire about personal involvement during radiation therapy**

Dear Patient, You are being treated with radiation therapy in our clinic and we would like to ask for your opinion! Therefore, we have created a short questionnaire that we would like you to fill out. We hope to further improve our services for patients.

Age ☐☐

Female ☐ Male ☐

I am being radiated at: Breast ☐ Bones ☐ Other body part ☐

Have I been radiated before in my life: Yes ☐ No ☐

1. Before my first radiation therapy I was/am anxious:

Does not apply ☐ Does rather not apply ☐ Rather applies ☐ Applies ☐

2. My concern about radiation therapy was/is greater than for other cancer-related therapies (e.g. operation, chemotherapy):

Does not apply ☐ Does rather not apply ☐ Rather applies ☐ Applies ☐

3. I use relaxation techniques before or during radiation therapy:

Does not apply ☐ Does rather not apply ☐ Rather applies ☐ Applies ☐

4. I would like to be guided on how to best relax before and during radiation:

Does not apply ☐ Does rather not apply ☐ Rather applies ☐ Applies ☐

5. I would be willing to take extra time to learn relaxation exercises:

Does not apply ☐ Does rather not apply ☐ Rather applies ☐ Applies ☐

6. Relaxation exercises would be especially helpful for me…

☐ In the treatment room

☐ Before radiation therapy in the waiting room

☐ At home

7. I would be interested in the following relaxation techniques:

☐ Autogenic training

☐ Progressive muscle relaxation

☐ Yoga

☐ Breathing exercises

☐ Dream journey/meditation

☐ Relaxation music

8. I would use an MP3 player with relaxation exercises and breathing exercises before and during radiation therapy:

Does not apply ☐ Does rather not apply ☐ Rather applies ☐ Applies ☐

9. I would take advantage of an exercise program in a small group:

Does not apply ☐ Does rather not apply ☐ Rather applies ☐ Applies ☐

10. I would like to be involved in the treatment and actively contribute to the best possible success of radiation therapy:

Does not apply ☐ Does rather not apply ☐ Rather applies ☐ Applies ☐

11. I would like to be guided by the medical staff on how I can contribute to the best possible success of radiation therapy:

Does not apply ☐ Does rather not apply ☐ Rather applies ☐ Applies ☐

Thank you very much for your time!
